# Supplementary material for: COVID-19 vaccine hesitancy among non-refugees and refugees in Kenya
Source: PLOS Glob Public Health. 2022 Aug 24;2(8):e0000917. doi: 10.1371/journal.pgph.0000917 (PMC10021684; doi:10.1371/journal.pgph.0000917)
Supplement: S1 Appendix — (DOCX) [file pgph.0000917.s001.docx]

S1 Appendix: Scaling of Information and Misinformation Variables

The variables used to create the information score are as follow:

- The use of masks in public would reduce the risk of contracting COVID-19 (1 being disagree, 2 being neutral, 3 being agree)
- Washing hands or using hand sanitizer reduces the risk of contracting COVID-19 (1 being disagree, 2 being neutral, 3 being agree)
- Keeping at least two meters/ two arms length distance from others reduces the risk of contracting COVID-19 (1 being disagree, 2 being neutral, 3 being agree)
- People can get COVID-19 from spending time in the same room as an infected person (0 disagree, 1 agree)
- COVID-19 can be dangerous to all age groups (0 disagree, 1 agree)

The information score was constructed by adding together all these variables (the lowest score being 3, the highest being 11). The inter-item correlation measured by cronbach’s alpha for these items was 0.7.

The variables used to create the misinformation score are as follow:

- Lemon and alcohol can be used as sanitizers against COVID-19 (0 disagree, 1 agree)
- Africans are immune to COVID-19 (0 disagree, 1 agree)
- COVID-19 does not affect children (0 disagree, 1 agree)
- COVID-19 cannot survive in warm weather (0 disagree, 1 agree)
- COVID-19 is just the common flu (0 disagree, 1 agree)
- Local herbs can be used to treat COVID-19 patients (0 disagree, 1 agree)
- People with a strong immune system do not need to work about COVID-19 (0 disagree, 1 agree)
- COVID-19 does not exist, it is a lie (0 disagree, 1 agree)
- Taking alcohol can make someone immune to COVID-19 (0 disagree, 1 agree)

The misinformation score was constructed by adding together these variables (the lowest score being 0, the highest being 9). The inter-item correlation measured by the Kunder-Richardson coefficient was 0.6.
