# Supplementary material for: COVID-19 vaccine hesitancy among non-refugees and refugees in Kenya
Source: PLOS Glob Public Health. 2022 Aug 24;2(8):e0000917. doi: 10.1371/journal.pgph.0000917 (PMC10021684; doi:10.1371/journal.pgph.0000917)
Supplement: S2 Appendix — (DOCX) [file pgph.0000917.s002.docx]

S2 Appendix: A Multivariable Logistic Regression Showing Odds of Being Vaccine Hesitant

|  | OR (95%CI) |
| --- | --- |
| Refugee | 1.76 (0.43,7.29) |
| Urban | 0.76 (0.48,1.2) |
| Refugee x Urban | 3.64 (1.47,9.01) |
| Age (Centered) | 0.99 (0.95,1.02) |
| Female | 1.04 (0.66,1.66) |
| Post-Primary Education | 0.32 (0.17,0.62) |
| Food Security | 0.85 (0.49,1.45) |
| Refugee x Food Security | 0.36 (0.1,1.29) |
| **Government Trust (Ref: Agree)** |  |
| Disagree | 8.66 (4.21,17.78) |
| Neutral | 4.53 (2.6,7.88) |
| Refugee x Disagree | 0.02 (0,0.13) |
| Refugee x Neutral | 0.14 (0.03,0.78) |
| Know's somebody with COVID-10 | 1.91 (0.81,4.51) |
| Refugee x Know's Somebody with COVID-19 | 0.12 (0.02,0.71) |
| Currently has Symptoms of COVID-19 | 0.55 (0.3,1.03) |
| Washes hands more because of COVID-19 | 0.11 (0.05,0.24) |
| Avoids handshakes because of COVID-19 | 0.29 (0.1,0.82) |
| Has not shopped in the past 14 days | 0.88 (0.51,1.51) |
| Avoids groups because of COVID-19 | 1.37 (0.63,2.98) |
| Used the internet in the past three months | 2.12 (1.13,3.97) |
| Refugee x Used the internet in the past three months | 0.21 (0.07,0.58) |
| Owns a Radio | 1.13 (0.44,2.92) |
| Owns a TV | 1.39 (0.82,2.35) |
| Refugee x Owns a TV | 0.15 (0.04,0.54) |
| Information Score (Centered) | 0.99 (0.73,1.34) |
| Refugee x Information Score | 0.57 (0.37,0.87) |
| Misinformation Score (Centered) | 0.96 (0.81,1.14) |
| Constant | 1.88 (0.36,9.72) |
