# Supplementary material for: COVID-19 vaccine hesitancy among non-refugees and refugees in Kenya
Source: PLOS Glob Public Health. 2022 Aug 24;2(8):e0000917. doi: 10.1371/journal.pgph.0000917 (PMC10021684; doi:10.1371/journal.pgph.0000917)
Supplement: Attachment — Submitted filename: Revision Responses.pdf [file pgph.0000917.s004.pdf]

Dear Editor,

Thank you for your consideration of our manuscript and for the feedback from the reviewers. Please see our responses to this feedback below.

<u>Reviewer 1</u>

1. Introduction: updata data on SARS CoV2 wordwilde. Furthermore introduce better the role of SARS CoV2 in your country and also as impact in health services. We know, from others experiences, how pandemic reduce the health services also for re-allocation of heatlworker and disruption of other services (for tb, malaria or maternal services), Introduce also this aspect as context. (see and cite Quaglio G, Tognon F, et al. Impact of Ebola outbreak on reproductive health services in a rural district of Sierra Leone: a prospective observational study. BMJ Open. 2019 Sep 4;9(9):e029093. doi: 10.1136/bmjopen-2019-029093.)
    a. We have included at the beginning of the introduction further context on COVID-19 vaccination nationwide, and the impact of low vaccination rates on mortality. We also include past evidence on vaccine hesitancy in Kenya, and mention at the end of the second paragraph of the introduction negative externalities of COVID-19 outbreaks, including the impact on healthcare services. However, as this is not a main goal of the paper we did not delve into this topic more in order to streamline the paper and save wordcount.
2. Discussion: discuss better and compare your results with other paper. Furthermore, the role of young is crucial in adherence in vaccination axpecially in Africa context where median age of population is young. (see and cite Marotta C, Nacareia U et al. Mozambican Adolescents and Youths during the COVID-19 Pandemic: Knowledge and Awareness Gaps in the Provinces of Sofala and Tete. Healthcare (Basel). 2021 Mar 13;9(3):321. doi: 10.3390/healthcare9030321.)
    a. We have in the discussion expanded upon the implications of our results regarding age, including referencing the Marotta et al paper. We also further compare our findings to past literature, including comparing our findings on government trust with those from high income countries.
3. Furthermore, give some global health proposal that came from your paper
    a. We throughout the discussion have added implications for policy, such as focusing on clinically at risk age groups or age groups at risk of transmission; and areas for further research, such as research into the impact on government trust; possible interventions; and research in other countries.

<u>Reviewer 2</u>

1. The data analysis of this study is strong. However, the introduction is superficial and does not provide an acceptable rationale for the study of vaccine hesitancy among refugees in Kenya. The authors do not present the picture of the pandemic in Kenya. No information on Covid-19 vaccine update is provided despite over 10.5 million Covid-19 vaccine doses administered in Kenya. The main finding from the study and its implication

are not discussed adequately. Conclusion section is inadequate. I have included additional major comments in the attached PDF.

    a. We have expanded on several points, including expanding the introduction to provide background on global vaccine uptake and refugees in Kenya; and expanding the discussion to include a stronger comparison the past literature. We expand on these points with your comments below.

2. The study lacks both a strong rationale for inclusion of, and detailed data on refugees.
    a. We have added into the introduction information on the refugee population in Kenya; information on why this population is important (given their size in Kenya and possible differences to the general population); and why it is important to analyse how refugees are different to the general population to differentiate care.
3. The study lacks both a thorough background and an organizing framework for examining Covid-19 vaccine hesitancy.
    a. How does this study build on prior studies?
        i. In the introduction we discuss the dearth of current literature and expand on this in the discussion.
    b. Given the low prevalence of COVID-19 in Kenya, why is it important to focus on vaccine hesitancy?
        i. In the introduction , we also mention why vaccine hesitancy is a particular concern in LMICs: the case fatality rate is more than double that in HICs, due to low vaccination rates
    c. Data from the WHo suggest that over 10.5 million doses of COVID-19 vaccine have been administered in Kenya as of 1/22. When were data collected, before or after roll out?
        i. We added in the introduction when data was collected and how this relates to vaccine role out in Kenya
    d. Why is it important to advance a study that is based solely on a hypothetical question?
        i. We have added information on the possible limitations of using a hypothetical questions, and the need for research to examine the impact of the use of a hypothetical question
    e. Given the binary nature of your data, maybe you should consider framing your narrative around vaccine refusal instead of hesitancy
        i. We respectfully disagree with this comment. Vaccine hesitancy is a spectrum of vaccine-making decisions and refusal lies along that spectrum (albeit, at one end of it). That is, refusal is a form of vaccine hesitancy and needs to be treated as such. Another form is delay, which while part of hesitancy is not refusal.
4. The main finding of the study seems to be that cynicism or public distrust in the government is a major driver of Covid-19 vaccine hesitancy in Kenya. However, very little discussion of this finding and its implications is provided by the authors.
    a. We have added discussion of this and further applicable literature to the discussion

Additional comments are embedded in the text BELOW.

b. The reason provided seems counterintuitive. I suspect that fear of overtly expressing distrust in a host government could instead lead the refugees to be docile and to conform to any Covid-19 vaccine mandates in the host country.
    i. We have added this as well as a possible reason.
c. Is an approach to problem-solving that gives priority to a small cross-section of the population most likely to resent public health efforts, the best way to use resources in resource-limited contexts like Kenya?
    i. Yes due to the still relatively large proportions of people who have these beliefs. We have added in numbers of these proportions.
d. This statement is confusing. How will research conducted in other countries and populations help elucidate the impact on Kenyan nationals of VH factors that were not investigated in your study?
    i. We have amended this statement to say research should also be conducted in different countries as contexts may differ.
