# Supplementary material for: COVID-19 vaccine hesitancy among non-refugees and refugees in Kenya
Source: PLOS Glob Public Health. 2022 Aug 24;2(8):e0000917. doi: 10.1371/journal.pgph.0000917 (PMC10021684; doi:10.1371/journal.pgph.0000917)
Supplement: Attachment — Submitted filename: VH among refugees and non-refugees in Kenya R2.docx [file pgph.0000917.s005.docx]

Dear Editorial Team and Reviewers,

Thank you for your comments on our paper. Please see our responses below:

**Editorial Comments**

1. Please amend your Financial Disclosure statement. If you did not receive any funding for this study, please simply state: “The authors received no specific funding for this work.”
   a. This has been done
2. Please update your Competing Interests statement. If you have no competing interests to declare, please state: “The authors have declared that no competing interests exist.”
   a. This has been done
3. Please provide a complete Data Availability Statement in the submission form. If your research concerns only data provided within your submission, please write “All data are in the manuscript and/or supporting information files.” as your Data Availability Statement.
   a. This has been done
4. Please include a separate legend for Figure 1 in your manuscript.
   a. This has been done
5. Please review your reference list to ensure that it is complete and correct. If you have cited papers that have been retracted, please include the rationale for doing so in the manuscript text, or remove these references and replace them with relevant current references. Any changes to the reference list should be mentioned in the rebuttal letter that accompanies your revised manuscript. If you need to cite a retracted article, indicate the article’s retracted status in the References list and also include a citation and full reference for the retraction notice.
   a. We have reviewed the citations and they are all correct.

**Reviewer 2 Detailed Comments**

**DATA & METHODS**

1. The strength of this paper probably lies in its data. It is suggested that authors provide links to data sources in the Data Availability section instead of sending readers to the References section.
   a. We have added a section in the methods with this link
2. There are inconsistencies between the comparator names in Table 1 (Kenyan vs Refugee) and Table 2 (Non-refugee vs Refugee). Please update as needed.
   a. We have throughout, including in the tables, updated to refugee and non-refugee
3. Related to the above point, IF POSSIBLE, it is strongly recommended that the authors provide a table of demographic characteristics for both groups. This may also be helpful to understand why they changed the group descriptor from “Kenyan” to “Non-refugee.”
   a. We have throughout, including in the tables, updated to refugee and non-refugee
4. I do not know what audience the authors had in mind while writing this paper, but it is always advisable to use a language that is accessible to the lay person, especially given that the chosen journal for dissemination is open access and prioritizes non-elitist and inclusive co-creation of knowledge. Are there ways to explain/describe concisely the technical terms in the Methods section (“unadjusted analysis”, “household weights”, “adjusted multivariate logistic regression”, “interaction”, “random forest model”, and “marginal effects”) so that the very refugee and non-refugee participants with above average levels of education may understand? In other words, can you re-write the second paragraph of the Methods such that it is easily understood by non-statistically savvy readers? In the same vein, consider adding legends to Figure 1 and the tables to explain their meanings or take-home message.
   a. We have throughout added parentheticals to explain these terms
5. Of note, stating that “Further information on the survey methodology has been published elsewhere” is not helpful to the reader.
   a. By including a link to the data source in the methods section we believe that this is now easily accessible and more helpful to the reader.

**KEY POINTS**

1. As implied in my comments above, while potentially more inclusive, the term “non-refugee” is nondescript and less informative than “Kenyan” natives. Be sure to provide a rationale for this choice of word in your Methods section.
   a. We added this explanation in the methods section to set the tone for the entire paper
2. Consider simplifying the phrase “weighted survey responses” with a less technical jargon.
   a. Where we mention weighted survey responses, we expand on this to say it is representative of the population of Kenya
3. Likewise, consider rephrasing the following sentencing so that it is understood by the average reader: “Interaction was seen between some factors and refugee status.”
   a. We explain in parentheticals that interactions are relationships which cause a change in the outcomes throughout
4. Related to the previous point, consider naming “some of the factors” that interact with refugee status.
   a. We have done this in the key points and abstract.

**ABSTRACT**

1. Please check for subject-verb agreement in the very first sentence of the abstract. “Factors associated … ~~is~~ (replace with ARE) poorly studied…”
   a. We have amended this
2. Please consider providing your definition of “vaccine hesitancy” (VH) after introducing this phrase in the abstract. E.g.: “…We estimated self-reported rates of VH as the percentage of ‘no’ responses to the question ‘Would you take the vaccine if offered to you at no cost?’ …”
   a. We have added this to the first line of the abstract
3. Related to the above point, do the same in your Methods section so that readers are clearly aware that the VH definition used in your study could be very different from that of the studies you cited in the Discussion section. No doubt the COVID-19 pandemic has significantly intensified research interest in VH. However, the lack of a common and consistent definition of VH conspires against public health advocates’ efforts to reduce misinformation and promote vaccine acceptance.
   a. We have added this to the methods, along with the exact question.
4. Correct the following sentence: “Binary response questions on misinformation and information into transformed into a scale.” (I assumed you wanted to write ‘…were transformed into…”).
   a. Corrected
5. As suggested earlier, please consider using less specialized jargon when describing your methodology and consider naming some of the variables that interact with refugee status.
   a. We attempt to explain this jargon in parentheticals
6. The implications from the study’s main finding are not well articulated. If refugees are found to be more COVID-19 vaccine accepting than the general Kenyan population, what does this means in light of the fact that vaccination coverage among refugee populations is often lower than that of host populations?
   a. We have added an additional sentence at the end of the abstract to better articulate the findings.

**INTRODUCTION**

1. Please consider nuancing the second sentence of the Introduction section by also mentioning effective COVID-19 treatment, not just high vaccine uptake rates, as a determinant of mortality rates.
   a. We have adjusted this to say low vaccine uptake is only a part of the reson
2. The sentence that starts with “Specifically, refugees and other refugee populations…” needs some corrections (Line 10, first Paragraph).
   a. We have corrected this
3. I don’t understand the basis of the following statement: “…hesitancy to the COVID-19 vaccines is of particular concern among refugee populations…” Do you have any supporting evidence for this statement? Given that your own study seems to suggest otherwise, why is it necessary or even desirable to suggest that VH is a concern among refugees? What about structural factors like xenophobia and racism?
   a. We have nuanced this to say that while it is an issue for both, it is very understudied among refugees
4. In line 9 of the second paragraph of the Introduction section you state: “Despite the large number of refugees in Kenya, little data exists on this on VH in this uniquely vulnerable population.” I am inclined to ask: How “uniquely vulnerable” are these refugees when compared to refugees living in other countries East African or Sub-Saharan African countries? I suggest removing the adverb “uniquely” from the sentence.
   a. We have removed this

**RESULTS**

1. The statement that urban-based refugees are more likely to be COVID-19 vaccine hesitant than rural-based counterparts whereas the opposite is true for non-refugee populations is intriguing and may need further development in the Discussion section.
   a. We have added a paragraph on this to the discussion
2. The next statement (3^rd^ paragraph; line 5) is too long and not very clear. The clause that starts after the colon (:) is not understandable.
   a. We have amended this sentence

**DISCUSSION**

1. First paragraph. It would be helpful to know how Orangi et al. as well as other investigators of VH in Sub-Saharan Africa have defined VH. If their operationalization and measurements of VH are different from yours, please inform the readers. This could very well explain the contrast in findings between the above studies and yours. Note: this observation applies to all the other studies cited in this paper. The main point here is that different definitions of VH will likely yield different study findings.
   a. Here and throughout we mention what different authors define as VH
2. The sentence in the second paragraph (beginning in line 10) that suggests that women and “young” people in the UK are more prone to VH needs some clarification about the age of the young. One could be led to speculate that “young” implies minors.
   a. We added the age range
3. Third paragraph (beginning in line 6). Authors state: “It seems plausible that behaviors operating under personal agency are linked to other variables associated with lower vaccine hesitancy.” While this sentence seems intuitive in appearance, it is confusing. VH is essentially about decision-making (or the lack thereof), a process that is largely nestled in personal agency. Is there really a need to further “link” personal agency variables to other determinants to reach a judgement of VH?
   a. We have amended this sentence to better explain our reasoning here
4. Authors further state: “We also found that those who use the internet are more likely to be vaccine hesitant, something hypothesized but not previously empirically demonstrated for the COVID-19 vaccine.” Are the authors not considering social media platforms as part of the Internet? If not, it will be helpful to explain the difference as it relates to the dissemination of viral disinformation/misinformation about vaccines. There are many published empirical findings on social media use and VH.
   a. We specify that this is not just social media, but the internet in general
5. Discussion of associations between trust in the government and VH would benefit from added clarification. Which “government” are we talking about? The same American respondents who distrust and even disdain the US Federal Government may often trust their local or state government, especially if the policies promoted by the latter undermine the effectiveness or enforcement of US Government vaccine policies. As suggested by the authors, the previous US administration politicized vaccination and undermined scientific expertise. However, this was not an isolated case. It has been speculated that in Tanzania, Kenya’s neighbor, the late president John Magufuli died from COVID-19 as he did not believe in the pandemic and actively encouraged Tanzanians to ignore social distancing measures. So, why is it then important to know ask respondents if they trust their government without first asking if the said government trusts science, public health, and vaccination?
   a. We clarify throughout that this is the 2021 Kenya government; and that it is important to understand what population’s understanding of the government’s views are
